# 3,5-Dinitro­benzyl methane­sulfonate

**DOI:** 10.1107/S1600536808020850

**Published:** 2008-07-12

**Authors:** Gul S. Khan, George R. Clark, David Barker

**Affiliations:** a Chemistry Department, The University of Auckland, Private Bag 92019, Auckland, New Zealand

## Abstract

The title compound, C_8_H_8_N_2_O_7_S, an inter­mediate in the synthesis of *N*,*N*-bis­(2-hydroxy­ethyl)-3,5-dinitro­aniline, exists as a discrete mol­ecule; the nitro groups are twisted with respect to the aromatic system [dihedral angles = 17.0 (1) and 26.3 (1)°].

## Related literature

For the utility of benzyl methane­sulfonates in synthesis, see: Barker *et al.* (2008 ▶); Bretonniere *et al.* (2004 ▶); Oh *et al.* (2004 ▶); Schirok *et al.* (2005 ▶). For the incorporation of *N*,*N*-bis­(2-hydroxy­ethyl)benzyl­amines in macromolecular metal complexes, see: Crans & Boukhobza (1998 ▶); Koizumi *et al.* (2005 ▶, 2007 ▶).
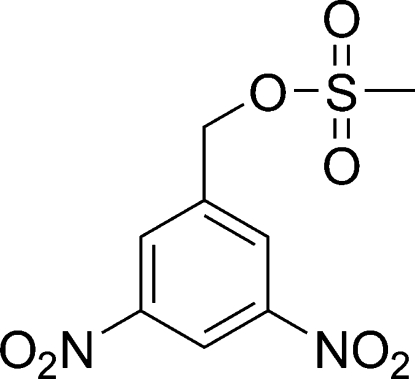


## Experimental

### 

#### Crystal data

C_8_H_8_N_2_O_7_S
*M* *_r_* = 276.22
Monoclinic, 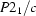

*a* = 9.3549 (5) Å
*b* = 8.7552 (5) Å
*c* = 14.1526 (8) Å
β = 107.430 (1)°
*V* = 1105.91 (11) Å^3^
*Z* = 4
Mo *K*α radiation
μ = 0.32 mm^−1^
*T* = 89 (1) K
0.32 × 0.14 × 0.14 mm

#### Data collection

Bruker SMART diffractometer
Absorption correction: multi-scan (*SADABS*; Sheldrick, 1997 ▶) *T*_min_ = 0.799, *T*_max_ = 0.971
6374 measured reflections
2233 independent reflections
1959 reflections with *I* > 2σ(*I*)
*R*_int_ = 0.019

#### Refinement

*R*[*F* ^2^ > 2σ(*F* ^2^)] = 0.033
*wR*(*F* ^2^) = 0.088
*S* = 1.06
2233 reflections
163 parameters
H-atom parameters constrained
Δρ_max_ = 0.28 e Å^−3^
Δρ_min_ = −0.49 e Å^−3^

### 

Data collection: *SMART* (Bruker, 1995 ▶); cell refinement: *SAINT* (Bruker, 1995 ▶); data reduction: *SAINT*; program(s) used to solve structure: *SHELXS97* (Sheldrick, 2008 ▶); program(s) used to refine structure: *SHELXL97* (Sheldrick, 2008 ▶); molecular graphics: *ORTEPIII* (Burnett & Johnson, 1996 ▶); software used to prepare material for publication: *SHELXTL* (Sheldrick, 2008 ▶).

## Supplementary Material

Crystal structure: contains datablocks I, global. DOI: 10.1107/S1600536808020850/ng2470sup1.cif

Structure factors: contains datablocks I. DOI: 10.1107/S1600536808020850/ng2470Isup2.hkl

Additional supplementary materials:  crystallographic information; 3D view; checkCIF report

## Figures and Tables

**Fig. 1.**
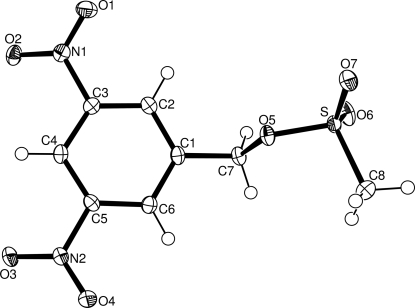
: Structure showing 50% probability displacement ellipsoids for non-hydrogen atoms and hydrogen atoms as arbitary spheres (Burnett & Johnson, 1996).

## supplementary crystallographic information

### Comment

Benzylic methansulfonates are readily prepared from benzylic alcohols and are often more easily prepared and more stable than the corresponding benzylic halide (Barker *et al.*, 2008). In particular benzylic methanesulfonates are useful for the preparation of *N*,*N*-bis(2-hydroxyethyl)benzylamines, which are nitrogen mustard precursors. The dual functionality of the two free hydroxyl groups along with a basic nitrogen have also seen *N*,*N*-bis(2-hydroxyethyl)benzylamines used in synthesis of numerous metal complexes including those containing vanadium (Crans & Boukhobza, 1998), manganese (Koizumi *et al.*, 2005, 2007) and iron (Koizumi *et al.*, 2005). There are no hydrogen bonding or π - π interactions in the crystal. The closest intermolecular contacts are O3 ··· N1 of 2.83 Å, and a pair of O ··· O 3.32 Å contacts between sulfonate oxygen atoms.

### Experimental

To a solution of 3,5-dinitrobenzyl alcohol (1.5 g, 7.57 mmol) and triethylamine (1.58 ml, 11.35 mmol) in dry THF (15 ml) at 0°C, under an atmosphere of nitrogen, was added dropwise a solution of methanesulfonyl chloride (0.88 ml, 11.35 mmol) in dry THF (5 ml) and the resulting solution stirred at room temperature for 3 h. The solvent was removed *in vacuo* and the residue diluted with ethyl acetate (150 ml), washed with brine (50 ml), dried (MgSO_4_) and the solvent removed *in vacuo* to afford the title compound (2 g, 96%) as a yellow solid which was recrystallized from ethyl acetate to give yellow crystals (m.p. 356–357 K) suitable for X-ray crystallography. IR νmax (NaCl)/cm^-1^ 3399, 1627, 1541, 1458, 1344. ^1^H NMR (400 MHz, CDCl_3_, δ, p.p.m.) 3.15 (3*H*, s, CH_3_), 5.40 (2*H*, s, CH_2_O), 8.60 (2*H*, br s, Ar—H), 9.05 (1*H*, br s, Ar—H). δ_C_ (100 MHz, CDCl_3_, δ, p.p.m.) 38.6 (CH_3_, CH_3_), 67.4 (CH_2_, CH_2_O), 119.5 (CH, Ar—C), 128.2 (CH, Ar—C), 138.6 (CH, Ar—C), 149.1 (quat., Ar—C). MS *m/z* (EI) 276 (*M*^+^, 1%), 197 (100), 181 (42), 134 (20). HRMS (EI) Found *M*^+^ 276.00489, C_8_H_8_N_2_O_7_S requires 276.00522.

### Refinement

Hydrogen atoms were placed in calculated positions and refined using the riding model [C—H 0.93–0.97 Å), with *U*_iso_(H) = 1.2 or 1.5 times *U*_eq_(C).

### Crystal data

**Table:**

C_8_H_8_N_2_O_7_S	*F*_000_ = 568
*M**_r_* = 276.22	*D*_x_ = 1.659 Mg m^−^^3^
Monoclinic, *P*2_1_/*c*	Mo *K*α radiation λ = 0.71073 Å
Hall symbol: -P 2ybc	Cell parameters from 4665 reflections
*a* = 9.3549 (5) Å	θ = 2.3–26.4º
*b* = 8.7552 (5) Å	µ = 0.32 mm^−^^1^
*c* = 14.1526 (8) Å	*T* = 89 (1) K
β = 107.430 (1)º	Rod, yellow
*V* = 1105.91 (11) Å^3^	0.32 × 0.14 × 0.14 mm
*Z* = 4	

### Data collection

**Table:**

Bruker SMART diffractometer	2233 independent reflections
Radiation source: fine-focus sealed tube	1959 reflections with *I* > 2σ(*I*)
Monochromator: graphite	*R*_int_ = 0.019
*T* = 89(1) K	θ_max_ = 26.4º
ω scans	θ_min_ = 2.3º
Absorption correction: multi-scan(SADABS; Sheldrick, 1997)	*h* = −9→11
*T*_min_ = 0.799, *T*_max_ = 0.971	*k* = −10→9
6374 measured reflections	*l* = −17→8

### Refinement

**Table:**

Refinement on *F*^2^	Secondary atom site location: difference Fourier map
Least-squares matrix: full	Hydrogen site location: inferred from neighbouring sites
*R*[*F*^2^ > 2σ(*F*^2^)] = 0.033	H-atom parameters constrained
*wR*(*F*^2^) = 0.088	*w* = 1/[σ^2^(*F*_o_^2^) + (0.0465*P*)^2^ + 0.6248*P*] where *P* = (*F*_o_^2^ + 2*F*_c_^2^)/3
*S* = 1.06	(Δ/σ)_max_ = 0.001
2233 reflections	Δρ_max_ = 0.28 e Å^−^^3^
163 parameters	Δρ_min_ = −0.49 e Å^−^^3^
Primary atom site location: structure-invariant direct methods	Extinction correction: none

### Special details

**Table:**

Geometry. All e.s.d.'s (except the e.s.d. in the dihedral angle between two l.s. planes) are estimated using the full covariance matrix. The cell e.s.d.'s are taken into account individually in the estimation of e.s.d.'s in distances, angles and torsion angles; correlations between e.s.d.'s in cell parameters are only used when they are defined by crystal symmetry. An approximate (isotropic) treatment of cell e.s.d.'s is used for estimating e.s.d.'s involving l.s. planes.
Refinement. Refinement of *F*^2^ against ALL reflections. The weighted *R*-factor *wR* and goodness of fit *S* are based on *F*^2^, conventional *R*-factors *R* are based on *F*, with *F* set to zero for negative *F*^2^. The threshold expression of *F*^2^ > σ(*F*^2^) is used only for calculating *R*-factors(gt) *etc*. and is not relevant to the choice of reflections for refinement. *R*-factors based on *F*^2^ are statistically about twice as large as those based on *F*, and *R*- factors based on ALL data will be even larger.

### Fractional atomic coordinates and isotropic or equivalent isotropic displacement parameters (Å^2^)

**Table:**

	*x*	*y*	*z*	*U*_iso_*/*U*_eq_	
S	0.28908 (4)	0.49088 (4)	0.39904 (3)	0.01554 (13)	
O1	0.79782 (14)	0.02166 (15)	0.65112 (9)	0.0229 (3)	
O2	0.75778 (13)	−0.16192 (14)	0.74420 (9)	0.0199 (3)	
O3	0.23786 (14)	−0.34566 (14)	0.65950 (9)	0.0230 (3)	
O4	0.06497 (14)	−0.21217 (16)	0.55662 (12)	0.0343 (4)	
O5	0.31800 (13)	0.32888 (13)	0.45017 (8)	0.0171 (3)	
O6	0.31513 (15)	0.60687 (14)	0.47320 (9)	0.0259 (3)	
O7	0.37522 (14)	0.49312 (14)	0.33100 (9)	0.0223 (3)	
N1	0.71506 (16)	−0.05791 (16)	0.68364 (10)	0.0166 (3)	
N2	0.19482 (16)	−0.23139 (16)	0.60944 (11)	0.0191 (3)	
C1	0.35300 (19)	0.15179 (18)	0.58246 (11)	0.0149 (3)	
C2	0.50631 (18)	0.11998 (19)	0.61537 (11)	0.0155 (3)	
H2A	0.5761	0.1957	0.6155	0.019*	
C3	0.55267 (18)	−0.02626 (19)	0.64782 (12)	0.0148 (3)	
C4	0.45474 (18)	−0.14488 (18)	0.64856 (11)	0.0148 (3)	
H4A	0.4880	−0.2418	0.6720	0.018*	
C5	0.30408 (18)	−0.10912 (18)	0.61204 (11)	0.0150 (3)	
C6	0.25038 (19)	0.03509 (19)	0.57931 (12)	0.0154 (3)	
H6A	0.1479	0.0536	0.5557	0.019*	
C7	0.29970 (19)	0.31110 (18)	0.54971 (12)	0.0163 (3)	
H7A	0.1955	0.3235	0.5470	0.020*	
H7B	0.3593	0.3862	0.5951	0.020*	
C8	0.0984 (2)	0.4858 (2)	0.33155 (17)	0.0319 (5)	
H8A	0.0702	0.5810	0.2974	0.048*	
H8B	0.0399	0.4696	0.3760	0.048*	
H8C	0.0807	0.4040	0.2843	0.048*	

### Atomic displacement parameters (Å^2^)

**Table:**

	*U*^11^	*U*^22^	*U*^33^	*U*^12^	*U*^13^	*U*^23^
S	0.0168 (2)	0.0125 (2)	0.0157 (2)	−0.00083 (14)	0.00236 (16)	0.00293 (14)
O1	0.0180 (6)	0.0286 (7)	0.0219 (7)	−0.0040 (5)	0.0057 (5)	0.0021 (5)
O2	0.0211 (6)	0.0168 (6)	0.0175 (6)	0.0029 (5)	−0.0006 (5)	0.0006 (5)
O3	0.0241 (7)	0.0172 (6)	0.0263 (7)	−0.0011 (5)	0.0057 (5)	0.0086 (5)
O4	0.0164 (7)	0.0248 (7)	0.0529 (9)	−0.0036 (5)	−0.0030 (6)	0.0140 (7)
O5	0.0237 (6)	0.0143 (6)	0.0134 (6)	0.0010 (5)	0.0055 (5)	0.0022 (4)
O6	0.0421 (8)	0.0133 (6)	0.0230 (6)	−0.0022 (5)	0.0105 (6)	0.0000 (5)
O7	0.0250 (7)	0.0227 (7)	0.0200 (6)	−0.0002 (5)	0.0079 (5)	0.0053 (5)
N1	0.0176 (7)	0.0164 (7)	0.0138 (6)	−0.0001 (6)	0.0018 (6)	−0.0035 (6)
N2	0.0189 (7)	0.0149 (7)	0.0227 (7)	−0.0007 (6)	0.0052 (6)	0.0035 (6)
C1	0.0207 (8)	0.0135 (8)	0.0100 (7)	0.0010 (6)	0.0038 (6)	0.0006 (6)
C2	0.0190 (8)	0.0147 (8)	0.0126 (7)	−0.0022 (6)	0.0044 (6)	−0.0009 (6)
C3	0.0155 (8)	0.0178 (8)	0.0099 (7)	0.0001 (6)	0.0018 (6)	−0.0016 (6)
C4	0.0200 (8)	0.0132 (8)	0.0101 (7)	0.0020 (6)	0.0030 (6)	0.0000 (6)
C5	0.0185 (8)	0.0140 (8)	0.0124 (7)	−0.0028 (6)	0.0046 (6)	−0.0004 (6)
C6	0.0171 (8)	0.0163 (8)	0.0120 (7)	0.0010 (6)	0.0031 (6)	0.0005 (6)
C7	0.0216 (8)	0.0136 (8)	0.0136 (8)	−0.0003 (6)	0.0053 (6)	0.0009 (6)
C8	0.0177 (9)	0.0331 (11)	0.0390 (12)	0.0005 (8)	−0.0002 (8)	0.0141 (9)

### Geometric parameters (Å, °)

**Table:**

S—O6	1.4279 (13)	C1—C7	1.507 (2)
S—O7	1.4290 (13)	C2—C3	1.385 (2)
S—O5	1.5783 (12)	C2—H2A	0.9300
S—C8	1.7538 (19)	C3—C4	1.387 (2)
O1—N1	1.2289 (19)	C4—C5	1.384 (2)
O2—N1	1.2322 (18)	C4—H4A	0.9300
O3—N2	1.2223 (18)	C5—C6	1.386 (2)
O4—N2	1.2323 (19)	C6—H6A	0.9300
O5—C7	1.4773 (19)	C7—H7A	0.9700
N1—C3	1.476 (2)	C7—H7B	0.9700
N2—C5	1.473 (2)	C8—H8A	0.9600
C1—C6	1.394 (2)	C8—H8B	0.9600
C1—C2	1.397 (2)	C8—H8C	0.9600
			
O6—S—O7	118.62 (8)	C5—C4—C3	115.40 (15)
O6—S—O5	109.52 (7)	C5—C4—H4A	122.3
O7—S—O5	105.51 (7)	C3—C4—H4A	122.3
O6—S—C8	109.88 (10)	C4—C5—C6	123.88 (15)
O7—S—C8	108.68 (9)	C4—C5—N2	117.80 (14)
O5—S—C8	103.51 (8)	C6—C5—N2	118.32 (14)
C7—O5—S	118.62 (10)	C5—C6—C1	118.68 (15)
O1—N1—O2	124.75 (14)	C5—C6—H6A	120.7
O1—N1—C3	117.64 (13)	C1—C6—H6A	120.7
O2—N1—C3	117.61 (14)	O5—C7—C1	105.58 (13)
O3—N2—O4	123.85 (14)	O5—C7—H7A	110.6
O3—N2—C5	118.35 (13)	C1—C7—H7A	110.6
O4—N2—C5	117.80 (14)	O5—C7—H7B	110.6
C6—C1—C2	119.55 (15)	C1—C7—H7B	110.6
C6—C1—C7	120.50 (15)	H7A—C7—H7B	108.8
C2—C1—C7	119.95 (15)	S—C8—H8A	109.5
C3—C2—C1	118.88 (15)	S—C8—H8B	109.5
C3—C2—H2A	120.6	H8A—C8—H8B	109.5
C1—C2—H2A	120.6	S—C8—H8C	109.5
C2—C3—C4	123.55 (15)	H8A—C8—H8C	109.5
C2—C3—N1	118.36 (14)	H8B—C8—H8C	109.5
C4—C3—N1	118.09 (14)		
